# Normal Bone Matrix Mineralization but Altered Growth Plate Morphology in the *Lmna^G609G/G609G^* Mouse Model of Progeria

**DOI:** 10.14336/AD.2024.1094

**Published:** 2025-11-04

**Authors:** Stéphane Blouin, Markus A. Hartmann, Nadja Fratzl-Zelman, Phaedra Messmer, Daniel Whisenant, Michael R. Erdos, Francis S. Collins, Maria Eriksson, Charlotte Strandgren, Wayne A. Cabral, Thomas Dechat

**Affiliations:** ^1^Ludwig Boltzmann Institute of Osteology at Hanusch Hospital of OEGK and AUVA Trauma Centre Meidling, Vienna, Austria.; ^2^Vienna Bone and Growth Center, Vienna, Austria.; ^3^Department of Biosciences and Nutrition, Karolinska Institute, Huddinge, Sweden.; ^4^Molecular Genetics Section, Center for Precision Health Research, National Human Genome Research Institute, National Institutes of Health, Bethesda, MD, USA.; ^5^Department of Oncology-pathology, Karolinska Institutet, Solna, Sweden.

**Keywords:** A-type lamins, Hutchison-Gilford progeria syndrome, bone matrix mineralization, quantitative backscattered electron imaging

## Abstract

Hutchison-Gilford progeria syndrome (HGPS) is a rare genetic disease caused by a mutation in *LMNA*, the gene encoding A-type lamins, leading to premature aging with severely reduced life span. HGPS is characterized by growth deficiency, subcutaneous fat and muscle issues, wrinkled skin, alopecia, and atherosclerosis. Patients also develop a bone phenotype with reduced bone mineral density, osteolysis and striking demineralization of long bones. To further clarify the tissue modifications in HGPS, we characterized bone mineralization in the *Lmna*^G609G/G609G^ progeria mouse model. Femurs from 8-week-old mice and humeri from 15-week-old mice were analyzed using quantitative backscattered electron imaging to assess bone mineralization density distribution, osteocyte lacunae sections and structural bone histomorphometry. Tissue sections were stained with Giemsa and Goldner trichrome for histologic evaluation. Bone tissue from *Lmna*^+/+^ and *Lmna*^G609G/G609G^ mice had similar mineral content at 3 different bone sites with specific tissue ages. The osteocyte lacunae features were not statistically different, but more empty lacunae were found in *Lmna*^G609G/G609G^ at both animal ages. Bone histomorphometry and histology demonstrated decreased bone volume per tissue volume in primary (8W: -23%, p=0.001; 15W: -38%, p=0.002) and secondary spongiosa (8W: -36%, p=0.001; 15W: -49 %, ns), as well as growth plate dysplasia with thinner unmineralized resting and proliferative zones in the *Lmna*^G609G/G609G^ mice versus controls (8W: -18%, p=0.006; 15W: -25%, p=0.001). Overall, the *Lmna*^G609G/G609G^ mouse develops chondrodysplasia with reduced trabecular bone volume. Mineral content findings at several tissue sites and ages suggest that bone dysplasia results from impaired bone formation with normal bone turnover.

## INTRODUCTION

Hutchinson-Gilford progeria syndrome (HGPS) is a rare genetic disorder with an estimated incidence of about 1 in 4-8 million births [1, 2]. It is most commonly caused by an autosomal dominant point mutation in the *LMNA* gene (c.1824C->-T, p.G608G in humans; c.1827C->-T, p.G609G in the mouse *Lmna* gene) [3, 4]. *LMNA* encodes A-type lamins, of which lamin A and C are the major isoforms, two ubiquitously expressed intermediate filament proteins and main components of the nuclear lamina, a filamentous meshwork that underlies the inner nuclear membrane [5, 6]. Lamin A is initially expressed as prelamin A and its post-translational processing comprises the addition of a farnesyl group to the cysteine residue of its C-terminal CAAX-box and a final cleavage step by the metalloprotease ZMPSTE24, removing the last 15 amino acids including the farnesylated cysteine [7]. The HGPS mutation leads to the activation of a cryptic splice site, resulting in the expression of an abnormal protein missing 50 amino acids near the carboxy terminus. Lacking the ZMPSTE24 cleavage site this protein, termed progerin, retains a farnesylated hydrophobic tail [3, 4, 8, 9].

Accumulation of progerin at the inner nuclear membrane causes progressive changes in nuclear architecture [10]. Although patients appear to have normal weight and appearance at birth, HGPS clinical features such as failure to thrive, delayed dentition, alopecia, skin abnormalities and lipodystrophy appear within the very first year of life [1, 2]. In addition, they eventually develop premature cardiovascular morbidities [11] and have a very limited life expectancy. According to the Progeria Research Foundation, patients with HGPS without treatment die from heart failure or stroke at an average age of 14.5 years (www.progeriaresearch.org/). At the cellular level, chronic activation of DNA damage checkpoints [12] and increased telomere attrition [13], result in premature cell senescence, consistent with HGPS being considered a premature aging disorder.

Laminopathies and other nuclear envelopathies are often associated with various bone abnormalities suggestive of possible comprehensive or locally disrupted mineralization processes [14]. For example, patients diagnosed with mandibuloacral dysplasia with lipodystrophy type A and B (MADA &MADB), due to mutations in *LMNA* or *ZMPSTE24* develop phalangeal, mandibular and clavicular osteolysis [15]. Restrictive dermopathy, most often caused by loss of ZMPSTE24 and subsequent ubiquitous accumulation of farnesylated prelamin A at the inner nuclear membrane, results also in resorption of clavicles and bone mineralization defects [14]. In HGPS severe osteopenia, dental demineralization and osteolysis in bone formed by membranous ossification have been reported from radiographic examination [16-18]. Gordon *et al*. pointed out the progressive nature of bone alterations in HGPS with onset only after birth with acroosteolysis being the earliest abnormal finding within a few months after birth, while clavicular and rib resorption start later in life [19]. Atypical demineralization of metaphyses and epiphyses was observed on X-rays in long bones, despite the diaphyses appearing normally mineralized [19]. Accordingly, bone mineral density measured by dual energy x-ray absorption in lumbar spine or total body of HGPS patients was reduced even after correction for height or bone age [20, 21]. These radiographic findings raise the question whether bone matrix mineralization is affected in HGPS or if the reduction in BMD is only due to a low bone volume.

To study the pathomechanism of progeria in more detail, several HGPS mouse models have been created, all of which develop similar features to the human illness, including a bone phenotype [14]. Absence of lamin A/C in *Lmna*^-/-^ mice induces osteopenia accompanied by a reduction in osteoblast and osteoclast number and function as well as in osteocyte number [22]. Interestingly, the bone phenotype is also obtained in skeletal muscle selective-*Lmna*^-/-^ mice showing a reduction in bone volume, especially in the spongiosa [23]. However, although no decrease in bone formation was found in these mice, elevated bone resorption was associated with increased osteoclastogenesis. Moreover, interleukin (IL)-6 levels were elevated in the mutant mice and knockout for IL-6 in this muscle selective-*Lmna*^-/-^ mice improved the bone phenotype [23]. Surprisingly, in osteoblast-specific *Lmna*^-/-^ mice no bone phenotype was observed. Zmpste24-deficient mice expressing only farnesylated pre-lamin A is another HGPS mouse model [24, 25]. Besides muscle weakness, these mice exhibit multiple fractures and osteolytic lesions along with low cortical and trabecular bone volume but normal bone turnover and osteoclast number. This mouse model simulates the situation in HGPS, as it presents a deficiency in mature lamin A and an accumulation of farnesylated pre-lamin A. Other HGPS-mouse models comprise the *Lmna*^Hg/+^ mice, which express solely progerin from one *Lmna*-allele and show osteolytic lesions [26], as well as the *Lmna*^L530P/L530P^ mice expressing a truncated and farnesylated mutant lamin A, which also display osteolytic lesions and a decrease in bone volume and bone mineral density [27, 28]. Another mouse model utilizes the expression of a human lamin A minigene carrying the HGPS mutation (c.1824C>T, p.G608G) in osteoblasts and odontoblasts. These mice also display bone and dental abnormalities, including defective mineralization and reduced osteoclast activity [29, 30]. Strikingly, mice that express progerin only in endothelial cells also develop a prominent bone phenotype, characterized by impaired osteogenesis and reduced bone volume [31]. The *Lmna^G609G/G609G^* mouse model, which is investigated in this study and reproduces the molecular lesion found in HGPS patients, presents with whole-body weight and size reduction [32, 33]. In addition, thinning of cortical bone and reduced trabecular bone volume were observed in tibiae and femurs with evidence of chondrodysplasia. Furthermore, skull size was reduced and the vertebral column distorted. Despite bone turnover seeming to be minimally affected by the mutation, treatment with tocilizumab, a neutralizing antibody raised against interleukin-6 receptors, partially rescued the reduction in bone volume [34].

It is not clear whether the observed bone volume alterations are associated with abnormal bone matrix mineralization. In addition, the report of empty osteocyte lacunae in HGPS mouse models with osteoblast-specific as well as endothelial cell-specific expression of progerin prompts consideration regarding the potential effects on osteocytes [30, 31]. Therefore, the aims of the current study were 1) to determine whether the degree of bone matrix mineralization and osteocyte lacunae characteristics are altered in HGPS and 2) to further characterize the previously observed alteration in the growth plate [33] and strengthen its characterization. Thus, we investigated long bones of *Lmna*^G609G/G609G^ mice at 8 and 15 weeks of age, timepoints representing pre- and post-skeletal maturity, which occurs at approximately 12 weeks in mice. Quantitative backscattered electron imaging (qBEI) was performed for precise mapping of the local mineral content. We used these images to determine the bone mineralization density distribution (BMDD) in three compartments that differ in tissue age (metaphysis, epiphysis, cortical midshaft) and to further evaluate size and shape of the osteocyte lacunae sections (OLS). Moreover, we took advantage of the qBEI images to evaluate the mineralized bone tissue volume and the characteristics of the growth plate. Given that noticeable differences in growth plate size and morphology were observed, we further performed histological analyses of growth plate development in this mouse model. We provide evidence that the endochondral bone phenotype in *Lmna*^G609G/G609G^ mice presents as a chondrodystrophy resulting from reduced bone formation and disregulated growth plate development.

## MATERIAL AND METHODS

### Mouse Strains and Animal Care

We investigated wild-type (*Lmna*^+/+^) and *Lmna*^G609G/G609G^ mice. The *Lmna*^G609G/G609G^ were obtained by mating the *Lmna*^G609G^ (c.1827C>T) knock-in mouse that expresses the murine endogenous *Lmna* gene harboring the corresponding mutation in classic HGPS patients. These mice originate from Carlos-Lopez Otín’s lab (Departamento de Bioquímica y Biología Molecular, University of Oviedo); their generation method and their genetic background has been published [32].

The investigated bone samples were provided by two different research facilities- the NIH and the Karolinska Institutet, respectively. From NIH, we obtained left femurs from animals aged 8 weeks: 20 *Lmna*^+/+^ (10 males & 10 females) and 18 *Lmna*^G609G/G609G^ (9 males & 9 females). From Karolinska Institutet, we obtained right humeri from animals aged 15 weeks: 7 *Lmna*^+/+^ (3 males & 4 females) and 7 *Lmna*^G609G/G609G^ (3 males & 4 females). *Lmna*^+/+^ and *Lmna*^G609G/G609G^ mice came from the same litters.

At NIH, the animal care and experiments were performed in accordance with a protocol approved by the NHGRI Animal Care and Use Committee. Mice were housed in individually ventilated cages with a 12-hour light/dark cycle. Standard NIH31 chow and water were provided ad libitum following weaning at 3 weeks of age. At Karolinska Institutet, experimental mice were housed in individually ventilated cages at a pathogen-free animal facility. Food and drinking water were provided *ad libitum*, and from postnatal week 3 softened mouse pellets were additionally provided on the cage floor. Animal studies at Karolinska Institutet were approved by the Linköping Ethical review board (Dnr: ID 429).

### Sample preparation for scanning electron microscopy

The femurs were directly fixed in 70% ethanol, whereas the humeri were fixed in 4% paraformaldehyde (pH 7.4) overnight at 4°C before the transfer to 70% ethanol for further conservation at 4°C. All samples were dehydrated with a solution series of increasing ethanol content (80%, 96%, and 100%), defatted with acetone and then embedded in polymethylmethacrylate (PMMA) [35]. For further handling, the samples were trimmed with a water-cooled low-speed diamond saw (Buehler Isomet 1000, Buehler Ltd., Lake Bluff, IL, USA) to form a block appropriate for further handling. Subsequently, histologic sections of approximately 3 µm thickness were prepared using a hard tissue microtome (Leica SM2500, Nussloch, Germany). The residual blocks were ground with a series of silicon carbide grinding papers (1200 and 2400 grit size) to expose longitudinal sections of mouse bone and then polished with a diamond suspension of 3 μm followed by 1 μm (Logitech PM5, Logitech Ltd., Glasgow, UK) prior to carbon coating (AGAR SEM carbon coater) to obtain a conductive surface necessary for scanning electron microscopy (SEM) observations.

### Quantitative backscattered electron imaging (qBEI)

For the quantification of bone matrix mineralization, the entire exposed bone sample area was imaged using SEM to obtain 8-bit gray scale images with a spatial resolution of 0.88 µm per pixel. Samples from the 8-week-old mice (20 *Lmna*^+/+^, 18 *Lmna*^G609G/G609G^) were measured using a SEM equipped with a tungsten hairpin cathode (Zeiss DSM 962) and samples from the 15-week-old mice (7 *Lmna*^+/+^, 7 *Lmna*^G609G/G609G^) were imaged using a field emission SEM (FESEM, Zeiss Supra 40, Oberkochen). Both microscopes are equipped with a four-quadrant semiconductor backscatter electron detector and were operated with an electron energy of 20 keV. The DSM was operated with 110 pA, 15 mm working distance, 90 s per image, 650x512 pixels while the FESEM was operated with 300 pA, 10 mm working distance, 90 s per image, 1024x768 resulting for both in around 250k electrons/µm². Prior to measurement, the SEMs were calibrated with standards of pure aluminum and carbon to allow for a conversion of measured gray levels into wt% calcium [35, 36].

### Bone mineralization density distribution (BMDD)

**Figure 1.**
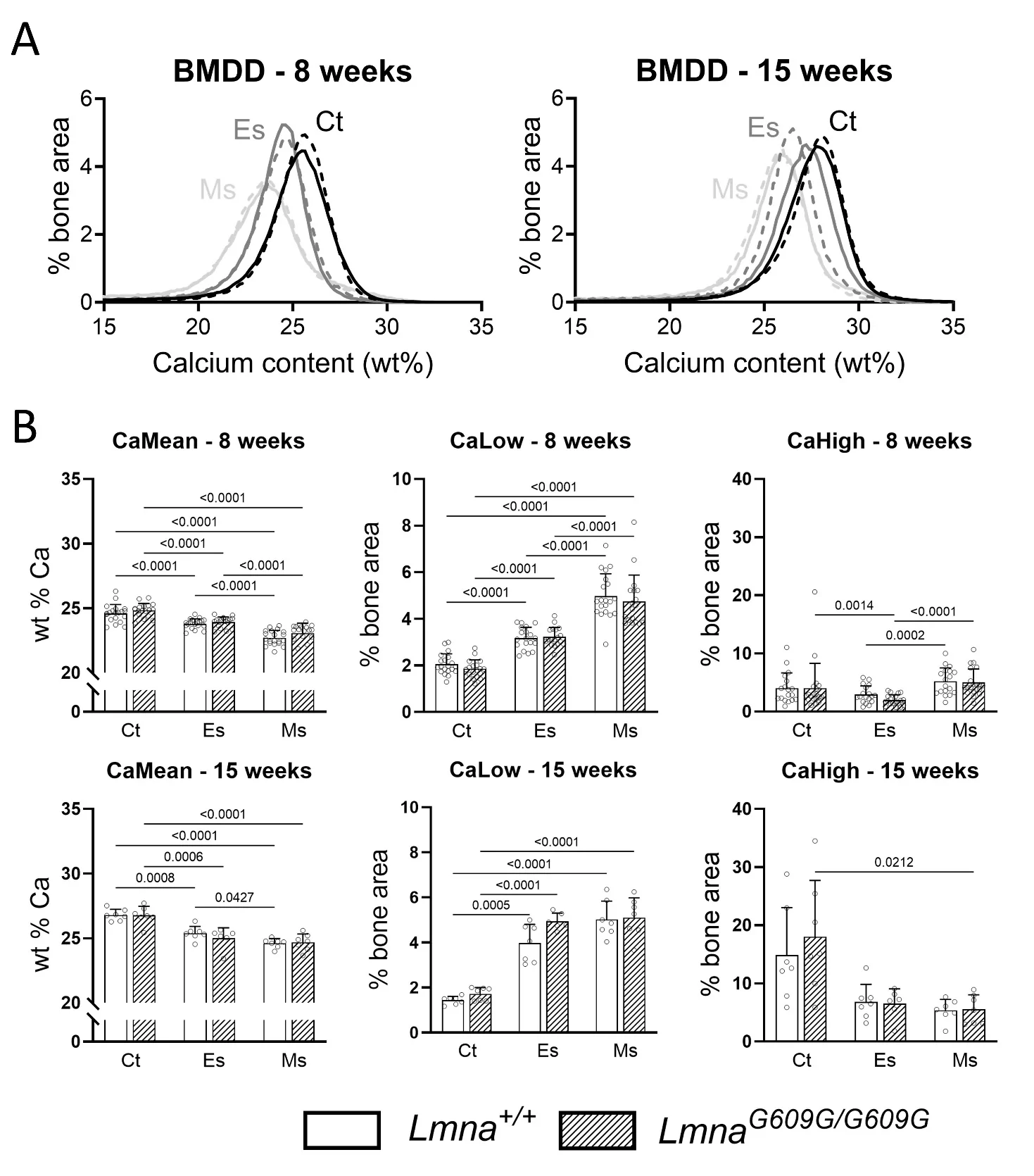
**Bone matrix mineralization results**. (**A**) Representative Bone Mineralization Density Distribution (BMDD) curves obtained with quantitative back scattered electron imaging on metaphysis (Ms), epiphysis (Es) and cortex (Ct) from *Lmna*^+/+^ (solid line) and *Lmna*^G609G/G609G^ (dashed line) mice. (**B**) Parameters CaMean, CaLow and CaHigh characterizing the BMDD. Percent calcium content by weight is denoted as wt %Ca. The data are presented as mean ± standard deviation. Significant differences based on post-hoc Tukey’s multiple comparisons tests after two-way ANOVA with site and genotype as factors are indicated. The BMDD and its parameters were obtained on (top) femurs from 8-week-old (20 *Lmna*^+/+^, 18 *Lmna*^G609G/G609G^) and (bottom) humeri from 15-week- old (7 *Lmna*^+/+^,7 *Lmna*^G609G/G609G^) mice. Male and female data were pooled. For numerical values, see Suppl. Table 1.

The bone mineralization density distribution (BMDD) is the frequency histogram of measured calcium content given in % of bone area. The histogram bin width of these BMDDs (i.e. the accuracy of the calcium content measurement) was 0.17 wt% Ca. We evaluated the BMDDs in 3 different regions per sample: cortical bone (Ct), epiphysis (Es) and metaphysis (Ms) for both 8-week-old (20 *Lmna*^+/+^, 18 *Lmna*^G609G/G609G^) and 15-week-old (7 *Lmna*^+/+^, 7 *Lmna*^G609G/G609G^) mice (see Fig. 1A). Five parameters were obtained from the BMDD curves:

CaMean = the weighted mean Ca-concentration of the measured bone area;

CaPeak = the position of the histogram peak indicating the most frequently measured calcium concentration;

CaWidth = the full width at half maximum of the distribution, indicative for mineralization heterogeneity;

CaLow = the percentage of lowly mineralized bone, i.e., the mineralized bone area having a calcium concentration below the 5th percentile of calcium concentration of a reference BMDD (using the metaphyseal BMDD of *Lmna*^+/+^ samples for each age as reference);

CaHigh = the percentage of highly mineralized bone areas, i.e., the bone area with a calcium concentration beyond the 95th percentile of calcium concentrations of a reference BMDD (using the metaphyseal BMDD of *Lmna*^+/+^ samples for each age as reference).

### Histomorphometry of mineralized bone tissue

**Figure 2.**
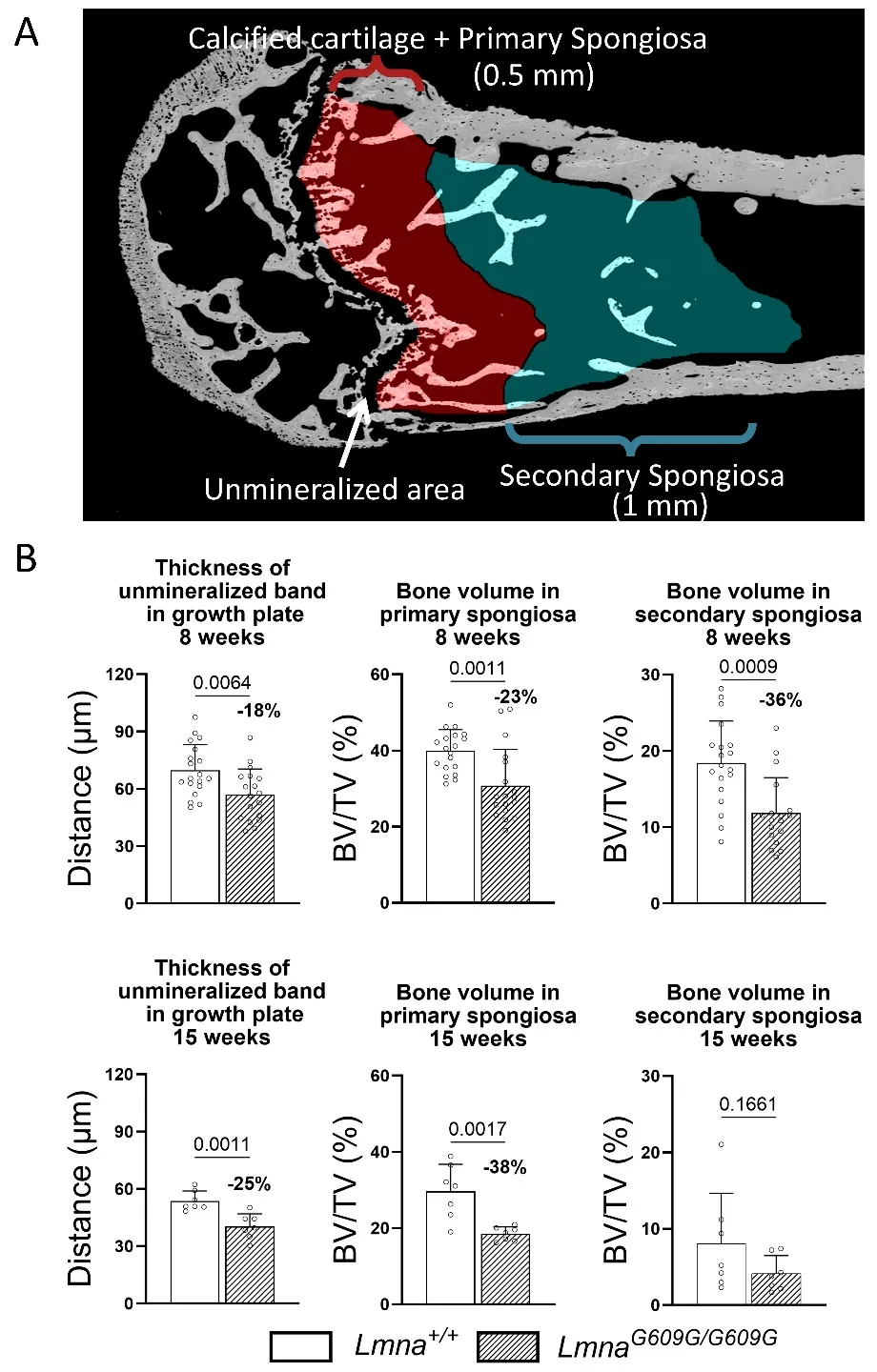
**Histomorphometry performed on mineralized tissue**. (**A**) Regions of analysis on an overview image of a femur obtained with qBEI. (**B**) Thickness of the unmineralized zone representing the resting and proliferative zone of the growth plate and volume fraction (bone volume per tissue volume - BV/TV) in the primary and secondary spongiosa. The data are presented as mean ± standard deviation. Significant differences based on student’s t-tests for unpaired data (or Mann-Whitney tests if normality was not verified) are indicated. The results were obtained on (top) femurs from 8-week-old (19 *Lmna*^+/+^, 18 *Lmna*^G609G/G609G^) and (bottom) humeri from 15-week-old (7 *Lmna*^+/+^,7 *Lmna*^G609G/G609G^) mice. Male and female data were pooled.

We took advantage of the images obtained with qBEI to perform a histomorphometric analysis of the mineralized regions (as non-mineralized regions appear black in a SEM analysis and cannot be distinguished from embedding material). Measurements were performed using an in-house custom-made macro for ImageJ (version 1.52n; NIH, Bethesda, MD, USA; https://imagej.nih.gov/ij/):

1) the bone volume/tissue volume (BV/TV), the trabecular thickness (Tb.Th) and trabecular number (Tb.N) in the first 500 µm from the mineralized region in the metaphysis close to the growth plate under the non-mineralized proliferative zone consisting of 1) the thin calcified cartilage on the bottom of the hypertrophic zone and 2) the primary spongiosa with mineralized cartilage spicules covered with new bone tissue (see Fig. 2A)
2) the BV/TV, Tb.Th and Tb.N of the secondary spongiosa resulting from the remodeling of the primary spongiosa in a region extending 1 mm after the 0.5 mm of the primary spongiosa (see Fig. 2A)
3) the thickness of the unmineralized part of the growth plate corresponding to the resting and proliferative zone (see Fig. 2A)

This analysis was performed on both 8-week-old (19 *Lmna*^+/+^, 18 *Lmna*^G609G/G609G^) and 15-week-old (7 *Lmna*^+/+^, 7 *Lmna*^G609G/G609G^) mice. Note that in one *Lmna*^+/+^ mouse, the trabecular area was not sufficient to perform a reliable measurement.

### Osteocyte lacunae sections (OLS)

We characterized in 2D the osteocyte lacunae sections (OLS) of the femurs (8-week-old: 19 *Lmna*^+/+^, 18 *Lmna*^G609G/G609G^) and humeri (15-week-old: 7 *Lmna*^+/+^, 7 *Lmna*^G609G/G609G^) based on six qBEI images from the cortex of the longitudinal sections containing an average of 253±59 lacunae in femurs and 425±133 lacunae in humeri. For this purpose, qBEI images were transformed into binary images using a fixed gray level threshold (5.2 wt% Ca) (for details see [37]). These binary images were analyzed for OLS using an in-house custom-made macro in the ImageJ software (version 1.52n; NIH, Bethesda, MD, USA; https://imagej.nih.gov/ij/). Subsequently, the OLS were extracted using size thresholds between 5 and 200 µm², respectively. Four parameters were obtained: (i) the OLS-porosity, (ii) the OLS-density (iii) the median OLS-area; (iv) the median OLS-perimeter (for details see [37]).

### Histology - growth plate

Three micrometer thin sections were cut from the undecalcified blocks with a hard tissue microtome (Leica SM2500, Nussloch, Germany), deplasticized with 2-methoxyethyl-acetate and stained with Giemsa and Goldner’s trichrome staining. Goldner’s staining allows to differentiate between mineralized bone (green) and osteoid (red), while Giemsa staining discriminates between cartilage (dark violet) and bone (light pink). Thus, Giemsa staining is especially suited to visualize the cartilage rich growth plate. Digital images were acquired with a Zeiss Axiophot light microscope (Zeiss, Oberkochen, Germany) equipped with a digital camera (AxioCam HRc, Zeiss) and the hypertrophic zone of the growth plate was measured (resolution of images 0.51 µm/pixel).

### Histology - osteocytes

In some of the mice from which bones were analyzed by qBEI, extra bone specimens were collected: at NIH, 4 *Lmna*^+/+^ (3 males and 1 female) and 5 *Lmna*^G609G/G609G^ (2 males and 3 females) right femurs from the 8-week-old animals; at Karolinska Institutet, 3 *Lmna^+/+^* (2 males and 1 female) and 3 *Lmna^G609G/G609G^* (2 males and 1 female) right femurs from 15-week-old animals. The samples were fixed in 4% paraformaldehyde and then decalcified at 4°C on a rocking table set on slow speed for approximately 3 weeks using 12.5% EDTA (pH 7.0). EDTA was changed every 3-5 days. Thereafter, the samples were dehydrated and subsequently embedded in paraffin wax. 4-5 µm tissue sections were cut from the blocks, mounted on Superfrost glass slides (Thermo Scientific) and incubated for 30 min at 60°C for adhesion of the sections onto the glass slides. The sections were further stained with Hematoxylin & Eosin according to a standard protocol including appropriate control slides.

Osteocyte lacunae number was determined for the entire diaphyseal cortical bone using a bright field light microscope. In accordance with previously published papers [29, 30], osteocyte lacunae were discriminated between lacunae containing cells, i.e. osteocytes showing well-defined and well-preserved nuclei, and empty lacunae containing no nuclei or a degenerated cell. Results were normalized against the total number of counted lacunae.

### Statistical analysis

The data are presented as mean ± standard deviation. Statistical analyses were carried out with GraphPad Prism 10.2.2 (GraphPad Software, Inc., La Jolla, CA, USA). Differences were considered statistically significant whenever p < 0.05. Normality of data was tested using the Shapiro-Wilk test since it is more powerful to detect nonnormality for small sample size [38].

For mineral content, male and female data were pooled. Two-way ANOVA with site and genotype as factors was performed for each age. As the statistical significance was achieved for the site, post-hoc Tukey’s multiple comparisons tests reducing type I errors were performed to identify which regions were different (Fig. 1A and Supplementary Table 1). To test for a possible sex effect, additional t-tests (not shown) between *Lmna*^+/+^ and *Lmna*^G609G/G609G^ mice of the same sex were performed but did not reveal any differences.

For histomorphometric data, student’s t-tests for unpaired data (or Mann-Whitney tests if normality was not verified) were performed on the pooled male and female data (Fig. 2). A possible sex effect was excluded by the same analyses done separately for each sex (Supplementary Fig. 2 and Supplementary Fig. 3).

**Figure 3.**
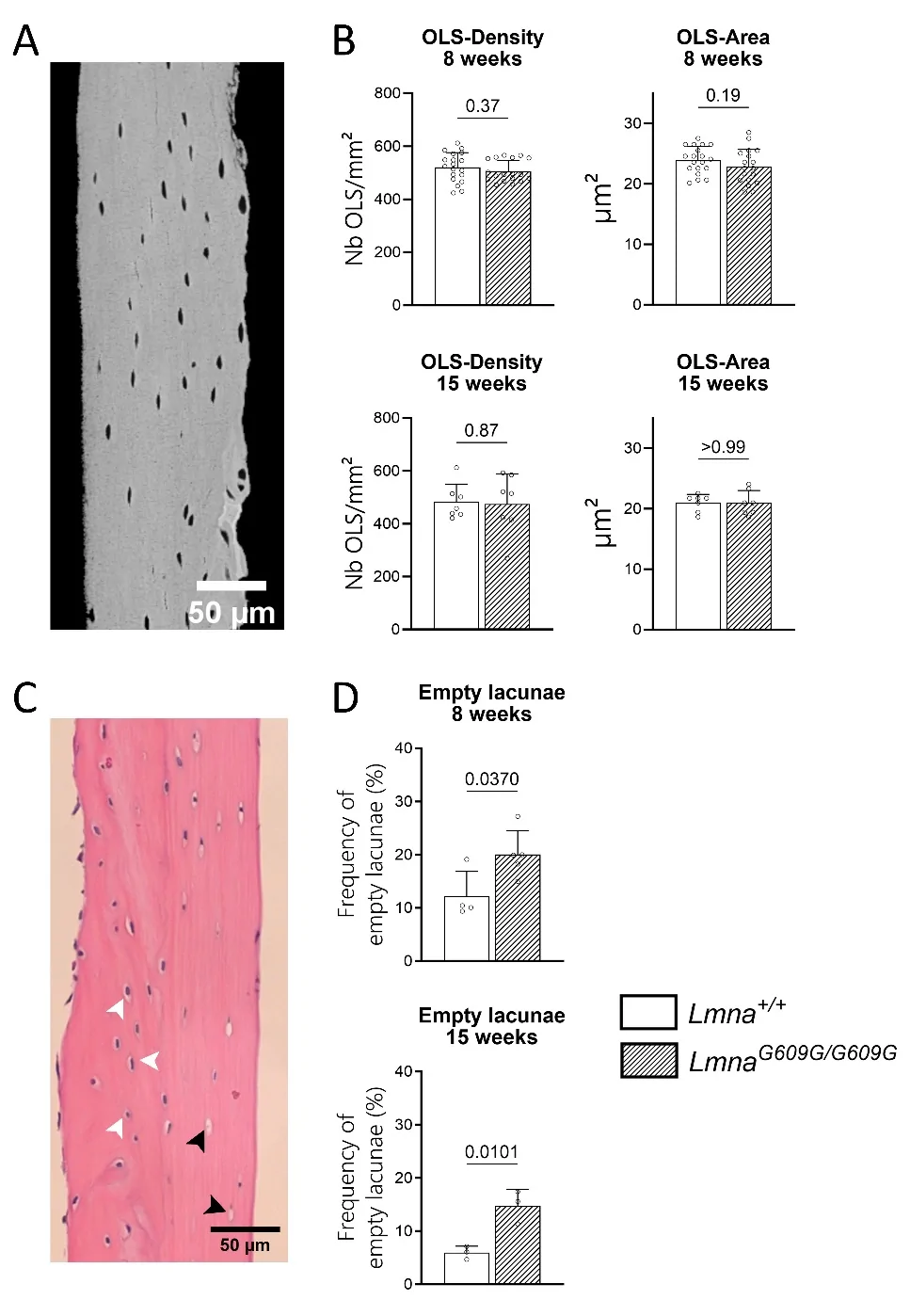
**Osteocyte lacunae**. (**A**) qBEI image of a representative femoral cortex with the unmineralized osteocyte lacunae section (OLS) (black) embedded in the mineralized bone tissue. (**B**) OLS results obtained from such images on femurs from 8-week-old (top) and humeri from 15-week-old (bottom) *Lmna*^+/+^ and *Lmna*^G609G/G609G^ mice. The data are presented as mean ± standard deviation. T-test for unpaired data (or Mann-Whitney tests if normality was not verified) showed no significant differences between groups. (**C**) Optical image of a section from a paraffin embedded demineralized sample stained with haematoxylin and eosin where empty lacunae (black arrowhead) and filled osteocyte lacunae (white arrowhead) are indicated. (d) Measurement of empty lacunae on femurs from (top) 8-week-old (4 *Lmna*^+/+^, 5 *Lmna*^G609G/G609G^) and (bottom) 15 week-old (3 *Lmna*^+/+^, 3 *Lmna*^G609G/G609G^) mice. The data are presented as mean ± standard deviation. Significant differences based on t-test for unpaired data are indicated.

For OLS data, t-test for unpaired data (or Mann-Whitney tests if normality was not verified) were performed on the pooled male and female data (Fig. 3) after possible sex effects were excluded.

For empty lacunae data, we choose to perform t-tests for unpaired data over non-parametric methods due to absence of suitable significance levels in very small size groups.

## RESULTS

### Overall bone matrix mineralization is not altered in Lmna^G609G/G609G^ mice

To investigate in detail if the degree of mineralization is impaired in HGPS, femurs from 8-week-old and humeri from 15-week-old *Lmna*^G609G/G609G^ mice were subjected to qBEI analyses and BMDDs of cortex, epiphysis and metaphysis were evaluated (Fig. 1 and Supplementary Table 1). In both age groups, we found a gradual shift towards lower mineralization density (CaMean and CaPeak) from cortical diaphyseal to epiphyseal and to metaphyseal bone (Fig. 1 and Supplementary Table1). In line with this finding, there is an increase in lowly mineralized bone area (CaLow) from cortical diaphyseal to epiphyseal and metaphyseal bone. For the highly mineralized bone area (CaHigh), a concordant decrease is found from cortical diaphyseal bone to epiphyseal bone. The similar CaHigh values in metaphyseal and cortical bone in 8-week-old mice can be explained by the presence of higher mineralized cartilage remnants from the growth plate in the metaphyseal bone. This is not observed in the bones of the older mice (15 weeks).

The overall degree of bone matrix mineralization, however, was not affected in *Lmna*^G609G/G609G^ mice when compared to bone from age-matched healthy littermates (Fig. 1 and Supplementary Table 1). At each evaluated age (8 and 15 weeks), the BMDD parameters were similar between *Lmna*^G609G/G609G^ and *Lmna*^+/+^ mice at the same bone sites (Ct, Es and Ms). Only CaLow was slightly affected in humeri from 15-week-old *Lmna*^G609G/G609G^ mice (see Supplementary Table 1). Thus, no significant differences in matrix mineralization of long bones were observed between progeroid mice and their wild-type littermates.

### Lmna^G609G/G609G^ mice have less bone volume in primary and secondary spongiosa and a thinner unmineralized zone in the growth plate

We used the obtained qBEI images to perform structural histomorphometry discriminating between i) the unmineralized area of the growth plate comprising the resting and the proliferative zone, ii) the mineralized cartilage in the hypertrophic zone covered with bone in the primary spongiosa and iii) the secondary spongiosa (shown in Fig. 2).

The measurements revealed that the thickness of the resting and proliferative zone in the growth plate of long bones of 8- and 15-week-old *Lmna*^G609G/G609G^ mice was significantly decreased (-18%, p=0.006 and -25%, p=0.001, respectively) compared to age-matched *Lmna*^+/+^ littermates (Fig. 2B).

In addition, we found a highly significant decrease in BV/TV in the primary spongiosa of *Lmna*^G609G/G609G^ bones compared to *Lmna*^+/+^ at both ages (-23%, p=0.001 and -38%, p=0.002, respectively) confirming the optical assessment of a thinner hypertrophic zone in few animals (Supplementary Fig 4). The Tb.Th was similar between *Lmna*^+/+^ and *Lmna*^G609G/G609G^ bones at both ages, but Tb.N was reduced in *Lmna*^G609G/G609G^ (Fig. 2B and Supplementary Table 2).

In the secondary spongiosa, a significant decrease in BV/TV (-36%, p<0.001) was observed in 8-week-old *Lmna*^G609G/G609G^ mice due to Tb.Th and Tb.N reductions in *Lmna*^G609G/G609G^ (-21%, p=0.001 and -21%, p=0.004, respectively). The same BV/TV and Tb.N trend was observed in 15-week-old mutant mice but did not reach significance (Fig. 2B), whereas Tb.Th was significantly reduced (-24%, p=0.01) (Fig. 2B and Supplementary Table 2). The examination of the data with respect to sex showed similar differences and trends in male and female mice (Supplementary Fig. 2 and Supplementary Fig. 3).

### Osteocyte lacunae number and shape are not altered in Lmna^G609G/G609G^ mice but empty lacunae are increased

The qBEI images obtained from mouse bones were used to perform OLS analysis. No statistically significant differences could be observed between HGPS mice and their wild-type littermates for any of the parameters describing the OLS in either the 8- or 15-week-old mice (Fig. 3B). It should be noted, however, that the qBEI method cannot discriminate between lacunae which harbor viable osteocytes and those that do not. Therefore, we observed histological sections of decalcified cortical bone (Fig. 3C) and counted on average about 1400 lacunae per 8-week-old animal and 2300 lacunae per 15-week-old animal. The proportion of empty lacunae was significantly increased in *Lmna^G609G/G609G^* in both 8- and 15-week-old mice (Fig. 3D).

**Figure 4.**
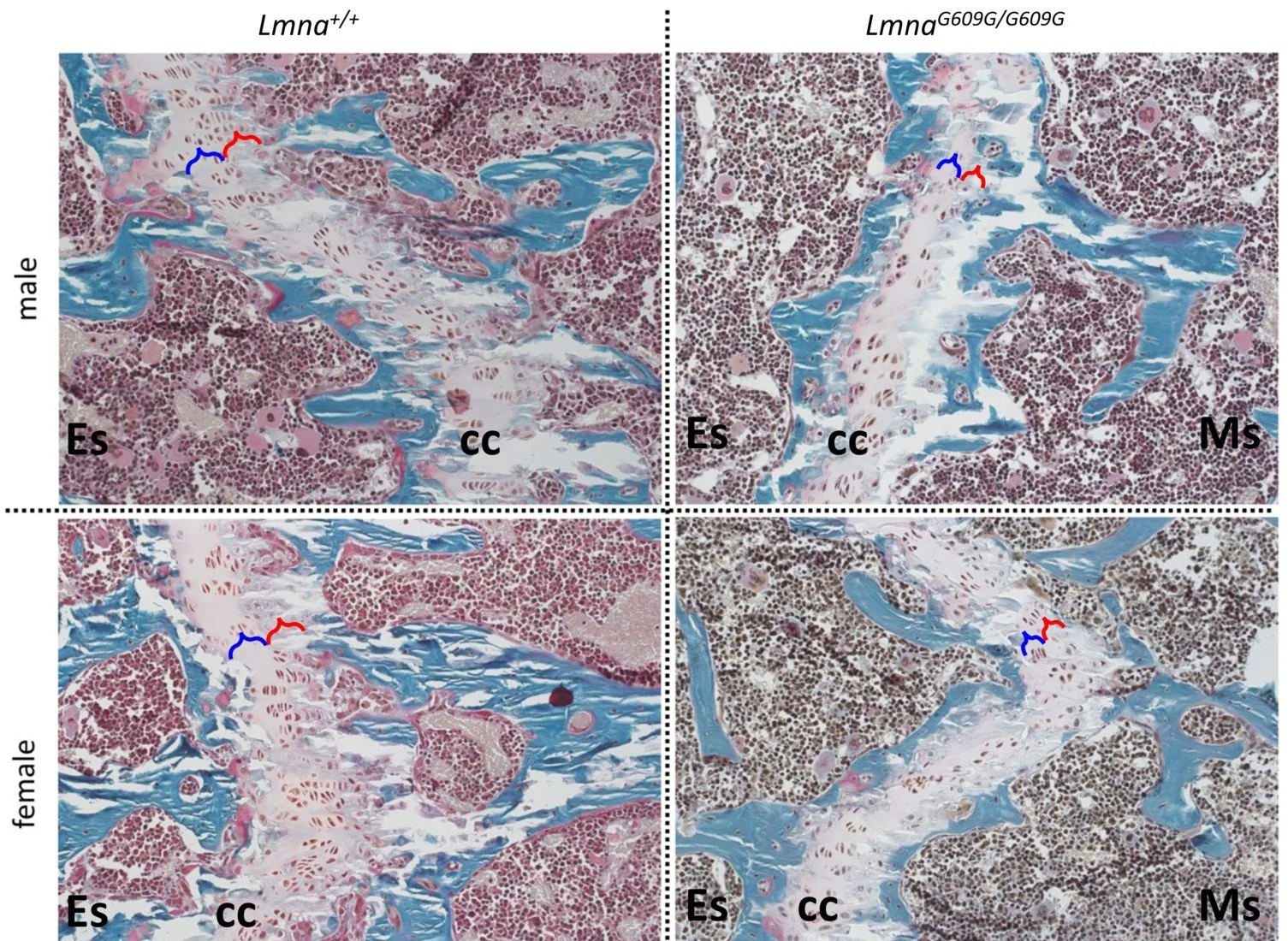
**Histological sections stained with Goldner’s trichrome showing the growth plate of the humeri of 15 weeks old *Lmna*^+/+^ and *Lmna*^G609G/G609G^ mice**. The mineralized bone tissue appears in blue/green, the non-mineralized bone tissue appears in red, the mineralized cartilage appears in light green and the non-mineralized cartilage is not stained. The epiphysis (Es) is located to the left of the growth plate and the metaphysis (Ms) to the right. In both *Lmna*^+/+^ and *Lmna*^G609G/G609G^ mice, a columnar proliferative zone and hypertrophic chondroblasts (cc) can be observed. However, both non mineralized (blue bracket) and mineralized cartilage (red bracket) appear to be thinner in *Lmna*^G609G/G609G^ mice compared to control. Images size = 354 µm x 265 µm.

### Growth plate is disturbed in *Lmna^G609G/G609G^* mice

The thinner, unmineralized zone corresponding to proliferative and resting zone in the growth plate of long bones of *Lmna*^G609G/G609G^ mice prompted our analysis of the cellular organization of the growth plate in more detail. Therefore, thin bone sections were stained with Goldner trichrome and Giemsa (Fig. 4 and Supplementary Fig 1). Histological evaluation showed that the growth plate appeared generally thinner in *Lmna*^G609G/G609G^ than in *Lmna*^+/+^ mice. Although chondrocytes in both genotypes were arranged in typical columns, the zone of flat proliferative chondrocytes was thinner in the *Lmna*^G609G/G609G^ mice compared to *Lmna*^+/+^ animals. Similarly, the hypertrophic zone was thinner in the *Lmna*^G609G/G609G^ mice compared to *Lmna*^+/+^ animals (Supplementary Fig. 4).

## DISCUSSION

Acroosteolysis, mandibular and clavicular osteolysis [15, 16, 18] and atypical demineralization of long bones [19] have been reported in HGPS patients. Beyond these abnormalities, it is not clear whether bone matrix mineralization is also generally altered and may contribute to the pathomechanism of the disorder. In the present work, we investigated the bone matrix mineral content of trabecular and cortical bone in the *Lmna*^G609G/G609G^ mouse model of HGPS. This model presents with reduced bone mass, altered rib cage shape and spinal curvature, and delayed calvarial mineralization with increased craniofacial and mandibular cartilage content [33]. In addition, the *Lmna*^G609G/G609G^ mice present reduced BMD at two months of age, particularly in the spongiosa. One of the aims of the present study was to clarify whether the low BMD is due to low mineralization of the bone material or to reduced bone volume.

Our findings of a BMDD shift towards lower mineralization density from cortical diaphyseal to epiphyseal and to metaphyseal bone in the *Lmna*^+/+^ control mice reflects local tissue age (from old to young) and was already described for other mouse models [39-42]. The *Lmna*^G609G/G609G^ mice presented a similar mineralization shift with decreasing tissue age as the controls, and since no mineralization difference was observed between the genotypes, similar bone turn-over in both genotypes can be assumed. Conversely, no differences in serum C-terminal telopeptide of type I collagen (CTX), mineralizing surface, mineral apposition and bone formation rate at periosteal and endosteal femoral surfaces were reported in these animals [33]. Noteworthy, higher serum tartrate-resistant acid phosphatase (TRAcP) was found at two months of age (no data for older animals exist) [33] and our results show that the decrease of mineralized BV/TV is due to reduced trabecular number rather than trabecular thickness. Taken together, these data suggest that more calcified cartilage is resorbed by the TRAcP-rich chondroclasts at the osteochondro-junction, and thus less calcified spiculae are formed below the growth plate in *Lmna*^G609G/G609G^. However, once primary trabeculae are formed, turnover is similar in both mutant and control mice. In addition to similar turn-over, the equivalent bone matrix mineral content in *Lmna*^G609G/G609G^ and *Lmna*^+/+^ mouse bones also suggests that the process of mineralization is unaltered. However, *in vitro* studies showed that progerin expression impairs the mineral deposition capacity of osteoblasts [30, 31, 33, 43]. One explanation of this discrepancy might be that mineralization is delayed *in vitro* with progerin expressing cells but might eventually reach the same degree as control cells in prolonged cultures that more closely reflect *in vivo* conditions. To clarify this in detail, additional studies are needed.

Elongation of long bones in mammals occurs at the growth plate by endochondral ossification and involves osteoblasts as well as chondrocytes, both of which are derived from mesenchymal stem cells (MSCs) [44-46]. Recent studies have shown that A-type lamins play an important role in the differentiation of MSCs as well as in the differentiation, maturation and functional regulation of osteoblasts and chondrocytes [30, 43, 47-53]. Morphometric measurements revealed an unbalanced shortening of femur and tibia, termed rhizomelia, in 2-month-old *Lmna*^G609G/G609G^ mice supported by the histological observation of a thinner growth plate indicative of chondrodysplasia [33]. In the present work, we found an 18% thinner resting and proliferative zone of the cartilage in the growth plate. This is in accordance with a stronger TUNEL staining of growth plate chondrocytes shown in these mice [33]. The specific observation of the hypertrophic zone with optical microscopy indicated a thinner hypertrophic zone at 8 and 15 weeks of age. Similarly, the volume of primary spongiosa observed with qBEI comprising highly mineralized cartilage spicules covered with new bone was also lower in the *Lmna*^G609G/G609G^ mice, again suggesting a decreased hypertrophic zone. This is in line with a reduced expression of genes associated with early and late stages of osteoblast differentiation reported in this HGPS mouse model [33]. Impaired osteoblast differentiation might be responsible for the decrease in bone volume observed in the secondary spongiosa, which is comparable to the trabecular results obtained with microcomputed tomography in 2, 3 and 8 month-old mice of the same model [32, 33]. A similar decrease in bone volume fraction was also found in long bones and vertebrae of other HGPS mouse models, including the *Zmpste24*^-/-^ mouse [24, 52], the *Lmna*^-/-^ mouse [22], the *Lmna*^HSA^-cko mouse [23], the *Lmna*^L530P/L530P^ mouse [27, 28] and tetop-LA^G608G+^ mice [30, 31]. However, no defects in the growth plate were reported in these mouse models. Taken together, our findings suggest that the low BMD found in HGPS patients is most likely due to a low bone mass and not due to hypomineralization of the bone material.

The formation of correct growth plate architecture is highly coordinated and essential for long bone development [54, 55]. It involves several crucial processes such as cell migration, mechanosensing, chondrogenic differentiation, establishing an extracellular matrix, oriented cell division, establishing polarity, formation of chondrocyte columns and the transdifferentiation of chondrocytes to osteoblasts [56-59]. Since lamins play an important role in many of these processes [6, 48, 60, 61], it is evident that progerin expression might interfere with correct growth plate formation. However, which processes are affected and subsequently contribute to the observed phenotype is subject for future studies. For example, it has been shown that progerin expression can impair mitosis [8, 62] and extracellular matrix formation [28, 33, 63, 64]. In addition, it has recently been shown that cadherin is involved in the regulation of growth plate architecture [65] and that cadherin complexes can be effected by lamin expression and mutant lamins [66-68].

Another aspect of our work was the determination of the osteocytic phenotype in the *Lmna*^G609G/G609G^ mice. In healthy humans, there is a reduction in osteocyte density with age at least until reaching adulthood [37, 69]. In mice a similar decrease of osteocyte density with age, as well as an impairment of the osteocyte lacuno-canalicular network and altered lacunar shape, were reported [70, 71]. Furthermore, osteoblast and osteocyte-specific inducible transgenic expression in a mouse model of HGPS (tetop-LA^G608G+;^ Sp7-tTA^+^) revealed that the vast majority of the osteocyte lacunae in cortical bone were empty [29, 30]. Interestingly, mice exhibiting endothelial cell-specific expression of progerin (tetop-LA^G608G+^; Cdh5-tTA^+^) also showed an increase in empty osteocyte lacunae [31]. Consequently, we also investigated the osteocytic phenotype in the current work. Our method based on electron microscopy is not able to discriminate between empty and filled osteocyte lacunae. Nevertheless, the quantification of OLS is a good surrogate to characterize the osteocytic population. It is extremely useful to assess osteocyte lacunar density and evaluate their shape in human biopsies and mice [37, 42, 72, 73]. In the present work on *Lmna*^G609G/G609G^ mice with ubiquitous expression of progerin, we could not find any change in osteocyte lacunar number and shape compared to control suggesting that the transition from osteoblast to osteocyte is unaffected. However, our investigation on H&E stained demineralized bone samples revealed an increased number of empty osteocyte lacunae in *Lmna^G609G/G609G^* in both 8- and 15-week-old mice, similar to what is found during normal aging in mice [74] and humans [37, 69].

A limitation of this work is the use of the *Lmna^G609G/G609G^* mouse model which does not fully recapitulate the progeroid phenotype. However, many features such as post-natal disease onset, growth retardation, progressive weight loss, alopecia, sclerotic skin, loss of subcutaneous fat, dental anomalies and bone alterations including reduced bone volume and impaired mechanical properties have been described [75]. Since mice appear to be more resistant to progerin accumulation than humans, the homozygous *Lmna^G609G/G609G^* mouse model has been employed because it develops a severe progeroid phenotype earlier than heterozygous *Lmna^G609G/+^* mice, thereby more closely reflecting the situation in the progeria patients presenting with heterozygous mutations [32, 75]. Moreover, we performed our analysis only on femurs and humeri, both weight-bearing bones in rodents. There is possibly an additional bone phenotype in non-weight-bearing bones similar to the development of osteolysis in phalanges, clavicles and ribs of patients. Another drawback of this study is the limited longitudinal aspect of the study with only two time points at 8 and 15 weeks. Unfortunately, another additional later time points are not possible due to the reduced life span of this model. Although a large number of characteristics have been measured in this work, it is noteworthy to mention that no statistical adjustment for multiple comparisons has been performed. It appeared unnecessary since several parameters of each aspect (mineralization and the morphology) were highly significantly changed.

In conclusion, the long bones of the *Lmna*^G609G/G609G^ mouse model present a distinct chondrodysplasia evidenced by a reduction of the unmineralized and mineralized part of the growth plate, leading to a decrease in bone volume. However, the degree of mineralization of the bone matrix in pre- and post-pubertal mice was unaffected in trabecular and cortical bone of femurs and humeri suggesting that bone turnover and tissue age were not disturbed. Importantly, the similarity of osteocyte lacunae number and shape in *Lmna*^G609G/G609G^ and *Lmna*^+/+^ mice confirm that a large part of the osteoblastic function is not affected, although osteocyte survival changes indicate a possible premature aging. This is in contrast to recent *in vitro* data showing greatly impaired osteoblastic mineralization capacity [30, 31, 33, 43]. These data led to the assumption that the reduced mineralization found in HGPS patients by dual-energy X-ray absorptiometry (DXA) and peripheral quantitative computed tomography (pQCT), respectively, arose from impaired bone mineralization and/or a decrease in bone turnover [14, 21]. Our data however suggest that neither is the case, but that bone development is impaired leading to less, but normally mineralized bone. It cannot be ruled out that the organic bone matrix might be affected by progerin expression, as lamins play and important role in establishing the extracellular matrix [28, 33, 63, 64], and that this affects the individual bone quality, similar to what occurs in most types of the rare bone disease osteogenesis imperfecta [76]. Therefore, the organic bone matrix should be investigated in detail by Raman spectroscopy or second harmonic generation microscopy, for example, in the *Lmna*^G609G/G609G^ mouse model. In addition, we also confirm recent findings that HGPS might be more a chondrodysplasia than a skeletal dysplasia [33]. These new findings might lead to new treatment strategies; administration of anabolic agents such as teriparatide (a recombinant human parathyroid hormone) to enhance bone development, as is done in the treatment of osteoporosis and osteogenesis imperfecta might be considered [77, 78]. Furthermore, treatment with the anticonvulsant carbamazepine, which has recently been tested in mouse models for metaphyseal chondrodysplasia and osteogenesis imperfecta, [79, 80] might also be studied in the HGPS mouse model. It must be mentioned, that while carbamazepine was found to be beneficial for the treatment of chondrodysplasia, it had no effect in the osteogenesis imperfecta mice but was instead found to have a negative impact on bone development in control mice. Although increased fracture rate does not appear to be a problem in HGPS, improving bone and skeletal quality will also improve the quality of life of patients, especially taking into account that the average life span for individuals suffering from HGPS increased by more than five years (from 14.5 to 19.5) due to new treatment strategies [21, 81] (www.progeriaresearch.org/2024/10/09/mourning-the-loss-of-prf-ambassador-sammy-basso/).

The exact mechanisms by which progerin expression affects bone development, structure and quality still needs to be investigated. It is most likely a combination of many events, including deregulation of gene expression, epigenetic chromatin organization and mechanosensing. This might subsequently result in impaired generation, differentiation and function of bone cells. In addition, the influence of paracrine factors such as IL6 and microRNAs derived from cell types and tissues other than bone might contribute to the bone phenotype observed in HGPS [23, 31, 34].

## Supplementary Materials

The Supplementary data can be found online at: www.aginganddisease.org/EN/10.14336/AD.2024.1094.

## Data Availability

The raw/processed data required to reproduce these findings will be available publicly at https://creed.lbg.ac.at.
