# Supplementary material for: Normal Bone Matrix Mineralization but Altered Growth Plate Morphology in the LmnaG609G/G609G Mouse Model of Progeria
Source: Aging Dis. 2024 Nov 4;16(5):3204–18. doi: 10.14336/AD.2024.1094 (PMC12339082; doi:10.14336/AD.2024.1094)
Supplement: Supplementary file 1 [file AD-16-5-3204-s.pdf]

SUPPLEMENTARY DATA

Normal Bone Matrix Mineralization but Altered Growth Plate Morphology in the *Lmna*$^{G609G/G609G}$ Mouse Model of Progeria

Stéphane Blouin, Markus A. Hartmann, Nadja Fratzl-Zelman, Phaedra Messmer, Daniel Whisenant, Michael R. Erdos, Francis S. Collins, Maria Eriksson, Charlotte Strandgren, Wayne A. Cabral, Thomas Dechat

© 2024. Blouin S et al. Published online at http://www.aginganddisease.org/EN/10.14336/AD.2024.1094

SUPPLEMENTARY DATA

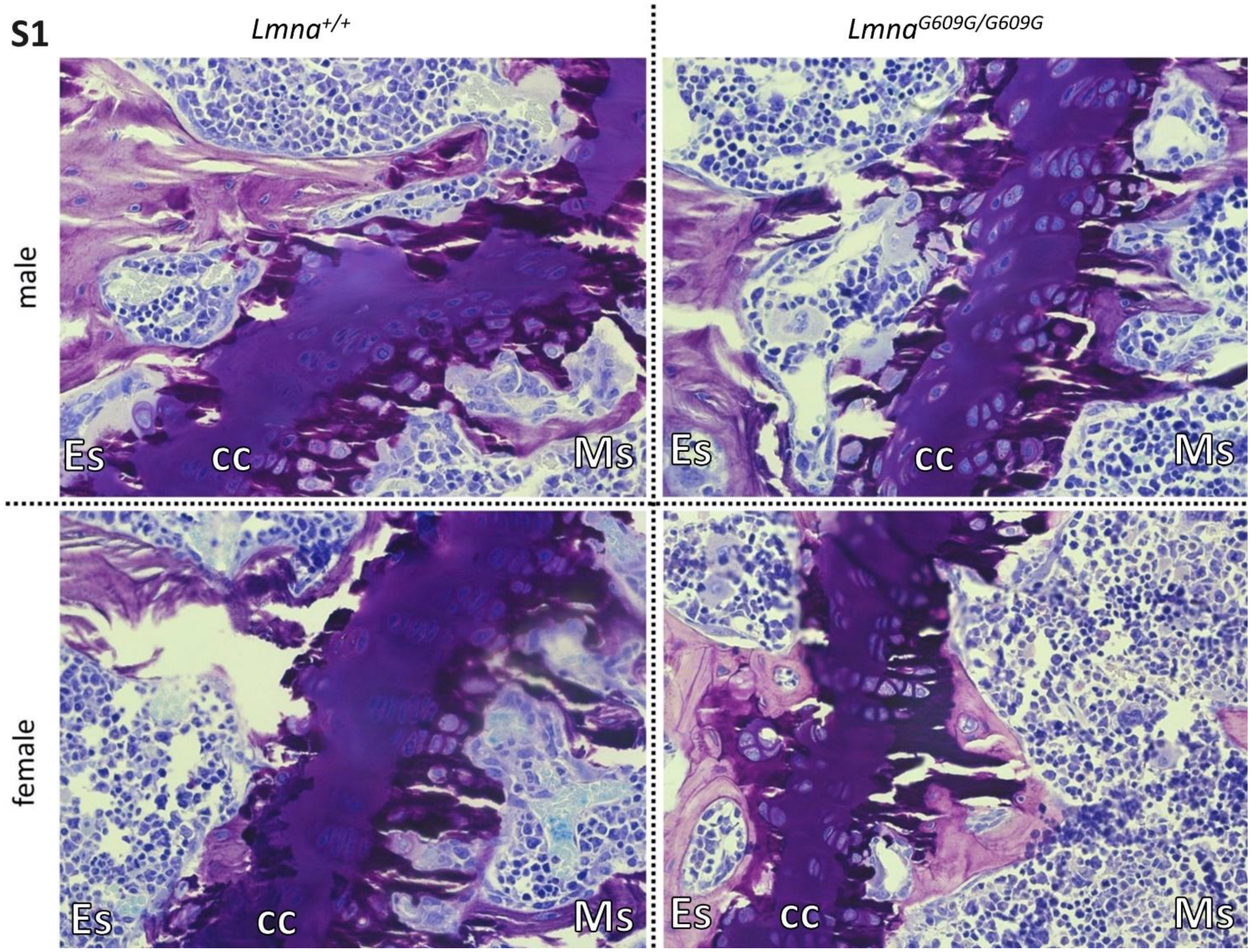

Supplementary Figure 1. Histological sections stained with Giemsa showing the growth plate of the humeri of 15 week-old *Lmna*$^{+/+}$ and *Lmna*$^{G609G/G609G}$ mice. The bone tissue appears in pink, the non-mineralized cartilage appears in light violet and the mineralized cartilage in dark violet. The epiphysis (Es) is located to the left of the growth plate and the metaphysis (Ms) to the right. In both *Lmna*$^{+/+}$ and *Lmna*$^{G609G/G609G}$ mice, a columnar proliferative zone and hypertrophic chondroblasts can be observed (cc). However, both non mineralized and mineralized cartilage regions are thinner in *Lmna*$^{G609G/G609G}$ mice compared to *Lmna*$^{+/+}$. Images size = 177 µm x 132 µm.

© 2024. Blouin S et al. Published online at http://www.aginganddisease.org/EN/10.14336/AD.2024.1094

SUPPLEMENTARY DATA

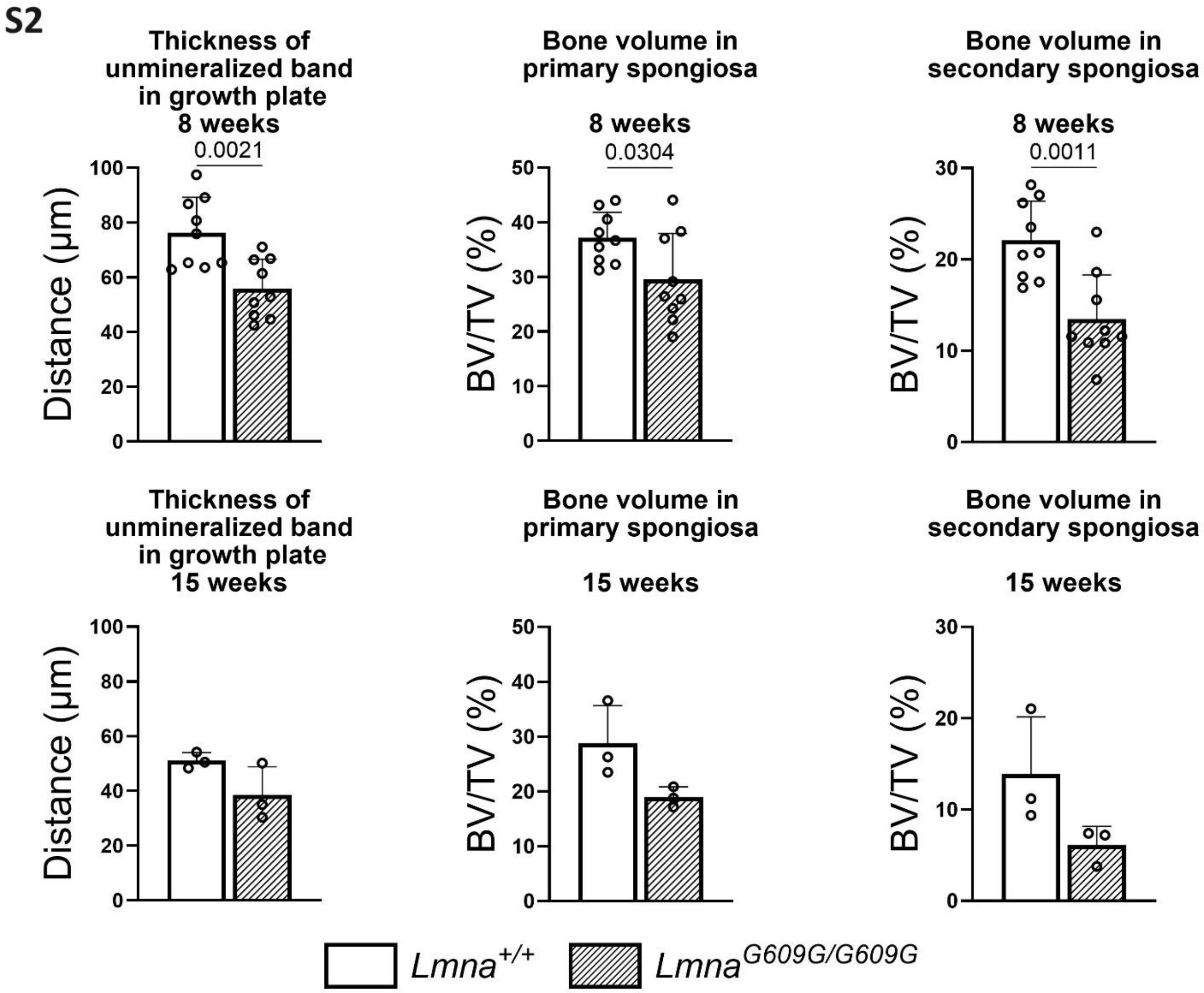

Supplementary Figure 2. Histomorphometry results in male *Lmna*$^{+/+}$ and *Lmna*$^{G609G/G609G}$ mice showing thickness of the unmineralized zone representing the resting and proliferative zone of the growth plate and volume fraction (bone volume per tissue volume - BV/TV) in the primary and secondary spongiosa. The data are presented as mean ± standard deviation. Significant differences based on student's t-tests for unpaired data (or Mann–Whitney tests if normality was not verified) are indicated. The results were obtained on (top) femurs from 8 week-old (9 *Lmna*$^{+/+}$, 9 *Lmna*$^{G609G/G609G}$) and (bottom) humeri from 15 week-old (3 *Lmna*$^{+/+}$, 3 *Lmna*$^{G609G/G609G}$) mice.

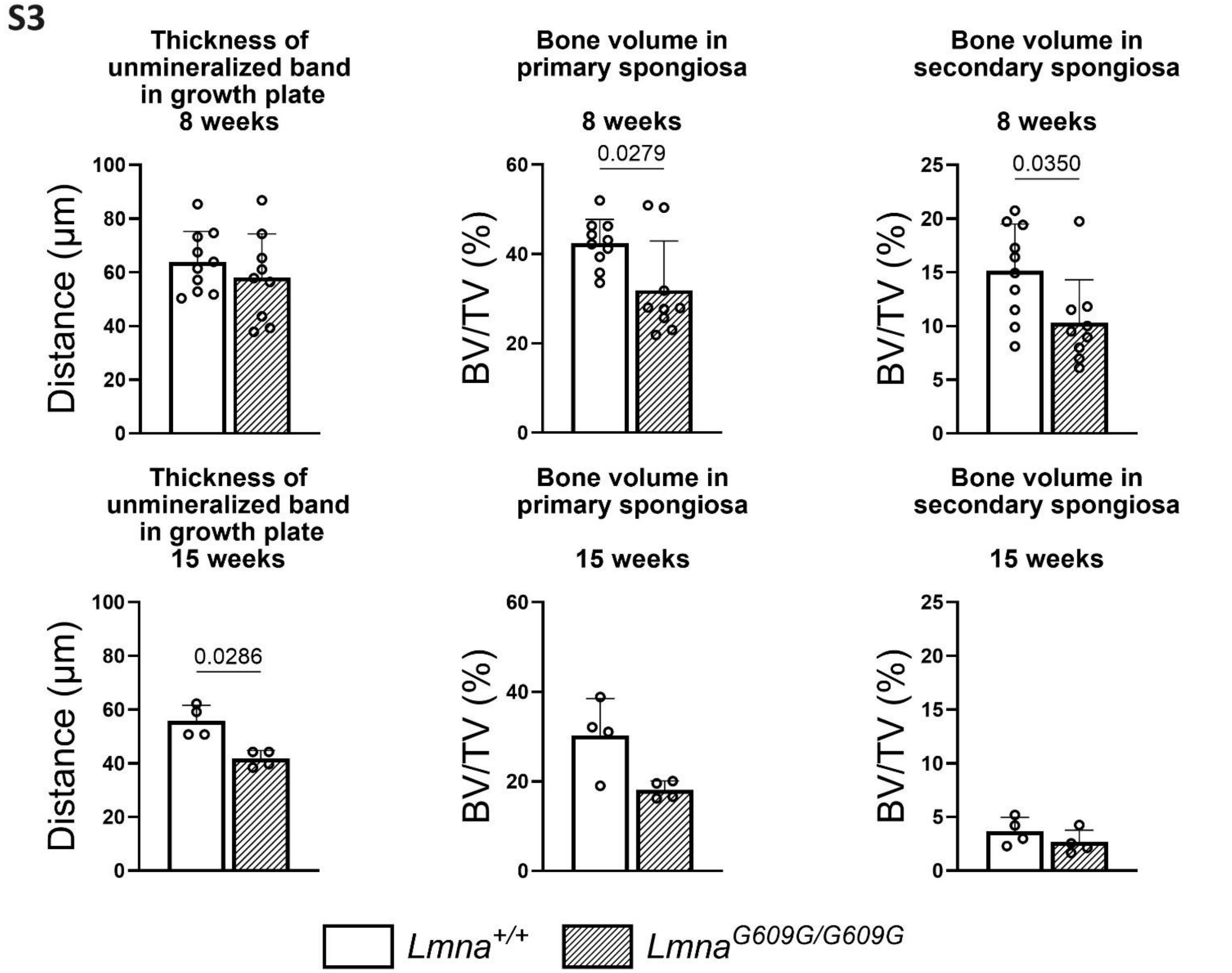

Supplementary Figure 3. Histomorphometry results in female *Lmna*$^{+/+}$ and *Lmna*$^{G609G/G609G}$ mice showing thickness of the unmineralized zone representing the resting and proliferative zone of the growth plate and volume fraction (bone volume per tissue volume - BV/TV) in the primary and secondary spongiosa.The data are presented as mean ± standard deviation. Significant differences based on student's t-tests for unpaired data (or Mann–Whitney tests if normality was not verified) are indicated. The results were obtained on (top) femurs from 8 week-old (10 *Lmna*$^{+/+}$, 9 *Lmna*$^{G609G/G609G}$) and (bottom) humeri from 15 week-old (4 *Lmna*$^{+/+}$, 4 *Lmna*$^{G609G/G609G}$) mice..

© 2024. Blouin S et al. Published online at http://www.aginganddisease.org/EN/10.14336/AD.2024.1094

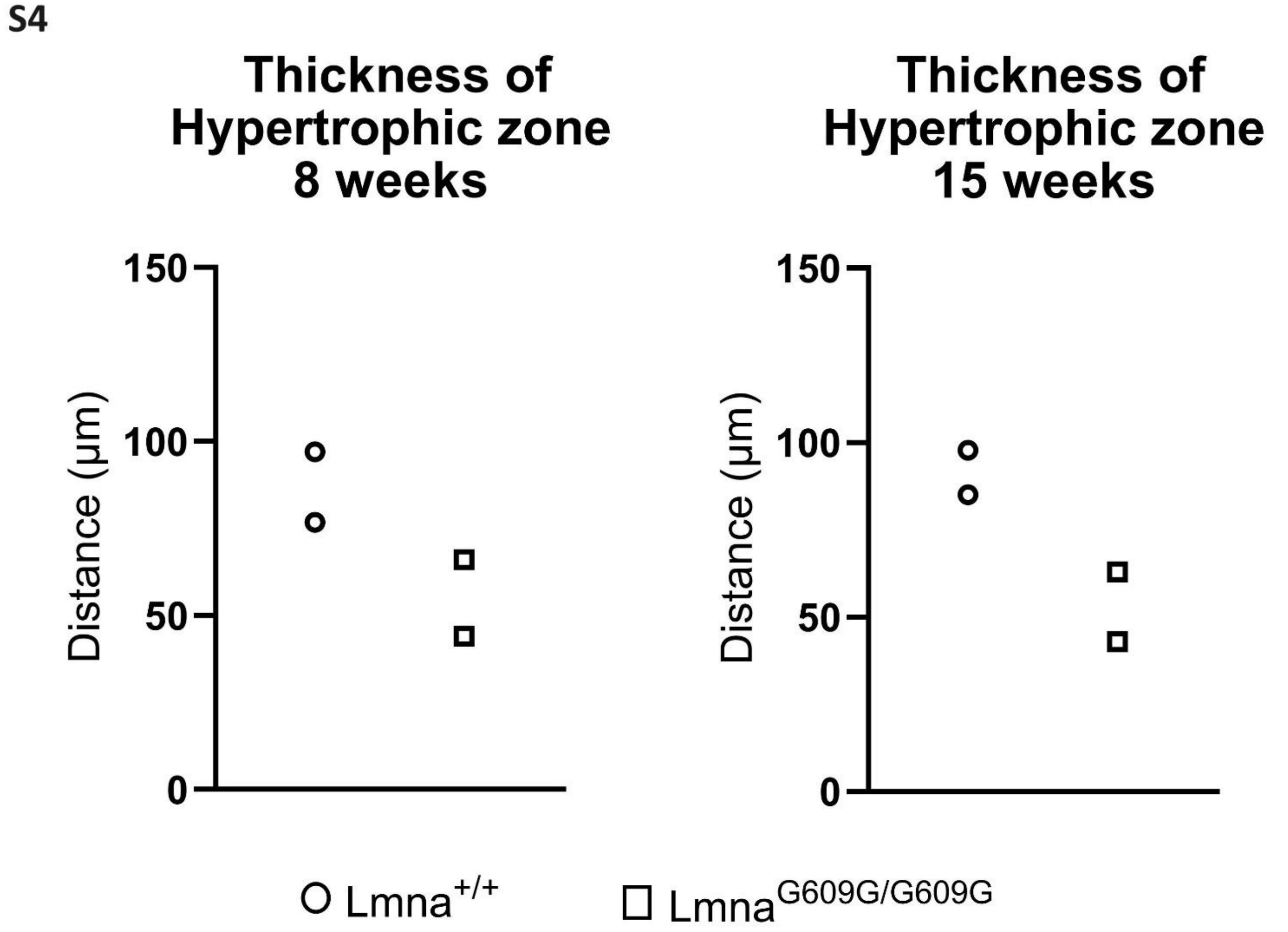

Supplementary Figure 4. Representative thickness measurement of the growth plate hypertrophic zone obtained on optical images from stained sections of undemineralized bone samples. The results were obtained on (left) femurs from 8-week-old (2 *Lmna*$^{+/+}$, 2 *Lmna*$^{G609G/G609G}$) and (right) humeri from 15-week-old (2 *Lmna*$^{+/+}$, 2 *Lmna*$^{G609G/G609G}$) mice.

© 2024. Blouin S et al. Published online at http://www.aginganddisease.org/EN/10.14336/AD.2024.1094

SUPPLEMENTARY DATA

Supplementary Table 1. Two-way ANOVA results with Post hoc-tests between the sites.

	Cortex		Epiphysis		Metaphysis		2-way ANOVA		
	Ctrl	Lmna^{G609G}	Ctrl	Lmna^{G609G}	Ctrl	Lmna^{G609G}	Interaction	Genotype	Site
**8 weeks-femur**									
**CaMean (wt% Ca)**	24.6 (0.68)	24.85 (0.52)	23.8 **** (0.38)	23.93 **** (0.40)	22.7 ****°°°° (0.57)	23.07****°°°° (0.77)	ns	ns	<0.0001
**CaPeak (wt% Ca)**	25.36 (0.66)	25.52 (0.50)	24.62 **** (0.35)	24.72 **** (0.34)	23.41 ****°°°° (0.56)	23.86****°°°° (0.66)	0.0162	ns	<0.0001
**CaWidth (Δ wt% Ca)**	3.32 (0.27)	3.23 (0.21)	2.82 **** (0.26)	2.89 **** (0.27)	3.99 ****°°°° (0.38)	3.76**°°°° (0.47)	ns	ns	<0.0001
**CaLow (% bone area)**	2.05 (0.44)	1.86 (0.38)	3.17 **** (0.47)	3.23 **** (0.41)	4.97 ****°°°° (0.96)	4.75****°°°° (1.13)	ns	ns	<0.0001
**CaHigh (% bone area)**	4.02 (4.3)	4.01 (2.64)	1.98 ** (0.91)	2.93 (1.48)	5.0 °°°° (2.32)	5.2°°° (2.31)	ns	ns	0.0005
**15weeks-humerus**									
**CaMean (wt% Ca)**	26.79 (0.44)	26.76 (0.71)	25.39*** (0.53)	25*** (0.8)	24.61****° (0.36)	24.68**** (0.65)	ns	ns	<0.0001
**CaPeak (wt% Ca)**	27.28 (0.4)	27.31 (0.71)	26.42* (0.47)	26.11** (0.93)	25.52****°° (0.37)	25.7**** (0.58)	ns	ns	<0.0001
**CaWidth (Δ wt% Ca)**	3.22 (0.17)	3.34 (0.38)	3.27 (0.23)	3.24 (0.57)	3.32 (0.32)	3.44 (0.35)	ns	ns	ns
**CaLow (% bone area)**	1.43 (0.18)	1.71 (0.29)	3.97**** (0.83)	4.94**** (0.36)	5.03**** (0.82)	5.1**** (0.88)	ns	0.0477	<0.0001
**CaHigh (% bone area)**	14.85 (8.21)	18.02 (9.7)	6.82 (3.03)	6.49 (2.56)	5.36 (1.89)	5.53° (2.52)	ns	ns	0.0004

Results are expressed as mean (SD)
Post-hoc test:
*p<0.05, **p<0.01,***p<0.001, ****p<0.0001 vs cortex from same genotype
°p<0.05, °°p<0.01,°°°p<0.001, °°°°p<0.0001 vs epiphyseal bone from same genotype

Supplementary Table 2. Histomorphometric results.

	Primary spongiosa			Secondary spongiosa		
	Lmna+/+	LmnaG609G/G609G	p value	Lmna+/+	LmnaG609G/G609G	p value
**8 weeks-femur**						
**BV/TV (%)**	39.92 (5.583)	30.75 (9.554)	0.0011	18.42 (5.505)	11.87 (4.618)	0.0009
**Tb.Th (µm)**	18.72 (4.863)	18.63 (3.996)	0.8	28.39 (6.568)	22.56 (5.138)	0.0011
**Tb.N (1/mm)**	22.15 (4.174)	16.7 (4.457)	0.0005	6.541 (1.628)	5.15 (1.059)	0.0042
**15weeks-humerus**						
**BV/TV (%)**	29.63 (7.095)	18.47 (1.826)	0.0017	8.045 (6.609)	4.138 (2.343)	0.2
**Tb.Th (µm)**	27.69 (5.603)	24.85 (2.055)	0.2	30.43 (6.098)	23.18 (1.954)	0.0112
**Tb.N (1/mm)**	10.73 (1.683)	7.475 (0.9416)	0.007	2.452 (1.597)	1.776 (0.9811)	0.4

Results are expressed as mean (SD).
Student's t-tests for unpaired data (or Mann–Whitney test if Shapiro–Wilk normality was not verified) were performed to test differences between *Lmna*$^{+/+}$ and *Lmna*$^{G609G/G609G}$. Statistically significant p values are in bold.

© 2024. Blouin S et al. Published online at http://www.aginganddisease.org/EN/10.14336/AD.2024.1094
